# Factors Contributing to Low Utilization of Intracoronary Imaging in Clinical Practice: A White Paper

**DOI:** 10.1016/j.jscai.2025.103607

**Published:** 2025-05-20

**Authors:** Javier Escaned, Marco Lombardi, Matthias Götberg, Nicolas Amabile, Adrian Banning, Emanuele Barbato, Salvatore Brugaletta, Shao-Liang Chen, Darshan Doshi, Bon-Kwon Koo, Ken Kozuma, Kalaivani Mahadevan, Dejan Milasinovic, Jan-Malte Sinning, Gabor Toth, Nieves Gonzalo, Mamas A. Mamas, Ajay J. Kirtane

**Affiliations:** a Hospital Clínico San Carlos IDISSC and CIBERCV, Complutense University of Madrid, Madrid, Spain; b Department of Internal Medicine and Medical Specialties (DIMI), Università di Genova, Genova, Italy; c Department of Cardiology, Skåne University Hospital, Lund University, Lund, Sweden; d Institut Cardiovasculaire Paris Sud, Massy, France; e Department of Cardiology and Oxford NIHR Biomedical Research Centre, Oxford University Hospitals, Oxford, United Kingdom; f Department of Clinical and Molecular Medicine, Sapienza University of Rome, Rome, Italy; g Hospital Clínic, Cardiovascular Clinic Institute, Institut d’Investigacions Biomèdiques August Pi i Sunyer (IDIBAPS), University of Barcelona, Barcelona, Spain; h Nanjing First Hospital, Nanjing Medical University, Nanjing, China; i Cardiovascular Research Center, Cardiology Division, Massachusetts General Hospital, Harvard Medical School, Boston, Massachusetts; j Department of Internal Medicine and Cardiovascular Center, Seoul National University Hospital, Seoul National University of College of Medicine, Seoul, South Korea; k Department of Cardiology, Teikyo University Hospital, Tokyo, Japan; l Department of Cardiology, Portsmouth Hospitals University NHS Trust, Portsmouth, United Kingdom; m Department of Cardiology, University Clinical Center of Serbia and Faculty of Medicine, University of Belgrade, Belgrade, Serbia; n Cellitinnen-Krankenhaus St. Vinzenz, Cologne, Germany; o Division of Cardiology, University Heart Center Graz, Medical University Graz, Graz, Austria; p Keele Cardiovascular Research Group, Centre for Prognosis Research Institute for Primary Care and Health Sciences, Keele University, Keele, Staffordshire, United Kingdom; q NewYork-Presbyterian Hospital/Columbia University Irving Medical Center, New York, New York; r Cardiovascular Research Foundation, New York, New York

**Keywords:** intracoronary imaging, intravascular ultrasound, optical coherence tomography, percutaneous coronary intervention

## Abstract

Intracoronary imaging (ICI) was introduced over 3 decades ago to complement conventional coronary angiography, yet its widespread uptake remains limited. This paper seeks to explore the potential causes behind low ICI utilization. The concepts of acceptability, acceptance, and adoption were applied to understand at which stage individual factors influence ICI implementation. Overall, the document aims at offering a comprehensive understanding of the challenges affecting ICI adoption, laying the foundation for effective change strategies. This approach is intended to address the broader, multifaceted nature of ICI implementation, providing a starting point for broadening its integration into clinical practice.

## Introduction

Intracoronary imaging (ICI) was developed more than 3 decades ago to complement conventional angiography and enhance the understanding of plaque and vessel morphology. The first intravascular ultrasound (IVUS) catheter delivering 2-dimensional images was developed in 1989,^1^ while light-based optical coherence tomography (OCT) for coronary use became available in 2001.^2^ Since its inception, ICI has been used for multiple purposes, including characterization of atheroma, understanding of vascular remodeling responses during atherogenesis, planning and guidance of percutaneous coronary intervention (PCI) to prevent restenosis and stent thrombosis, and assessment of plaque regression associated with pharmacologic therapy.^3^, ^4^, ^5^, ^6^, ^7^, ^8^, ^9^, ^10^, ^11^, ^12^, ^13^, ^14^, ^15^, ^16^, ^17^ Most recent meta-analyses of randomized controlled trials (RCTs) performed within the drug-eluting stent (DES) era have shown superior patient outcomes, including mortality benefit, when PCI is guided with ICI compared with angiography.^18^, ^19^, ^20^ Current clinical practice guidelines include recommendations for ICI in specific clinical and anatomical scenarios.^21^, ^22^, ^23^, ^24^, ^25^ Currently, there are at least 14 commercially available ICI systems (Supplemental Table S1). While all these seem to reflect a story of success for ICI, there are multiple indicators that, on a global scale, the uptake of ICI in clinical practice is very low, with high heterogeneity even at a country or individual hospital level.

While scientific societies have issued dedicated expert documents on ICI and with inclusion of dedicated sessions in scientific conferences is the norm,^26^, ^27^, ^28^, ^29^, ^30^, ^31^ there is a paucity of studies investigating the causes of low ICI utilization in clinical practice. This document aimed to provide an expert consensus on the barriers to ICI uptake and explore potential solutions. In contrast to existing clinical consensus documents, it did not intend to provide information on how to use ICI in clinical practice. Instead, it aimed to achieve a broader understanding of factors that influence ICI implementation, in the belief that this is a key starting point in formulating change strategies and for testing their effectiveness in real-world practice.

## Organizational aspects of this document

This was an investigator-initiated project launched by a group of interventional cardiologists with a track record in the field of ICI who were willing to explore the causes of low ICI utilization today. Two online and 1 in-person meetings led to the identification of key factors affecting ICI utilization, the preparation of a white paper, and the distribution of tasks.

One of the basic premises of this document is that the problem of ICI uptake is a multifaceted one and, thus, its analysis requires a broader consideration of factors generally not addressed in other fora. Former approaches have largely focused at a high level on a few variables, like device costs or reimbursement, which cannot explain the vast differences in ICI adoption within individual countries and institutions. This article seeks to understand more deeply how the utilization of ICI is modulated by factors that operate at the level of the interventional cardiologist, catheterization laboratory nurses and allied professionals (NAPs), hospital administrators, and health care policies. The document proposes actionable outcomes based on addressing each of the identified factors.

Our analysis of the factors that influence the uptake of ICI is based on concepts of acceptability, acceptance, and adoption. These 3 concepts have been used in investigating the success or failure of technology uptake.^32^ In this document, acceptability refers to the perception of ICI before its use by the interventional cardiologist. Acceptance is the interventionalist’s perception of ICI value and ease of use after an initial application in clinical practice. Finally, adoption refers to a multistage process that begins with the successful acceptability and acceptance of ICI by PCI operators and gets extended to other personnel involved in the interventional management of patients. Ultimately, it ends up with the commitment of hospital administrators and health care systems. In general, acceptability and acceptance are considered prerequisites for adoption of a new technology (Central Illustration).

**Central Illustration:**
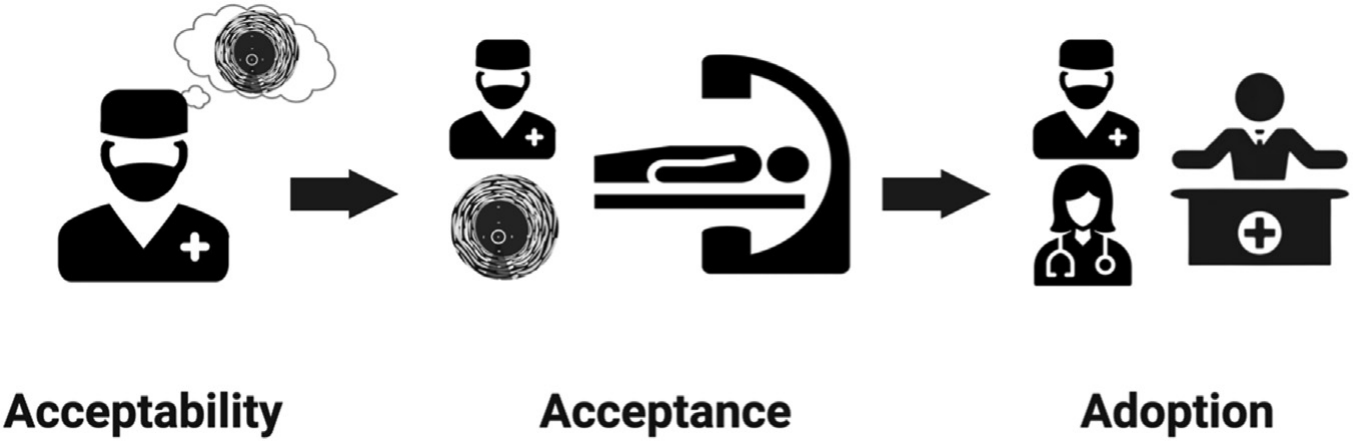
Graphical summary of the concepts of acceptability, acceptance, and adoption of intracoronary imaging (ICI). Acceptability refers to the perception of ICI before its use by interventional cardiologists. Acceptance is the perception of its value and ease of use after initial clinical practice. Adoption is a multistage process starting from successful acceptability and acceptance by percutaneous coronary intervention operators, extending to other interventional personnel and culminating in the commitment from hospital administrators and health care systems. Acceptability and acceptance are prerequisites for adoption.

## Available supporting evidence and current recommendations on ICI

**Figure 1:**
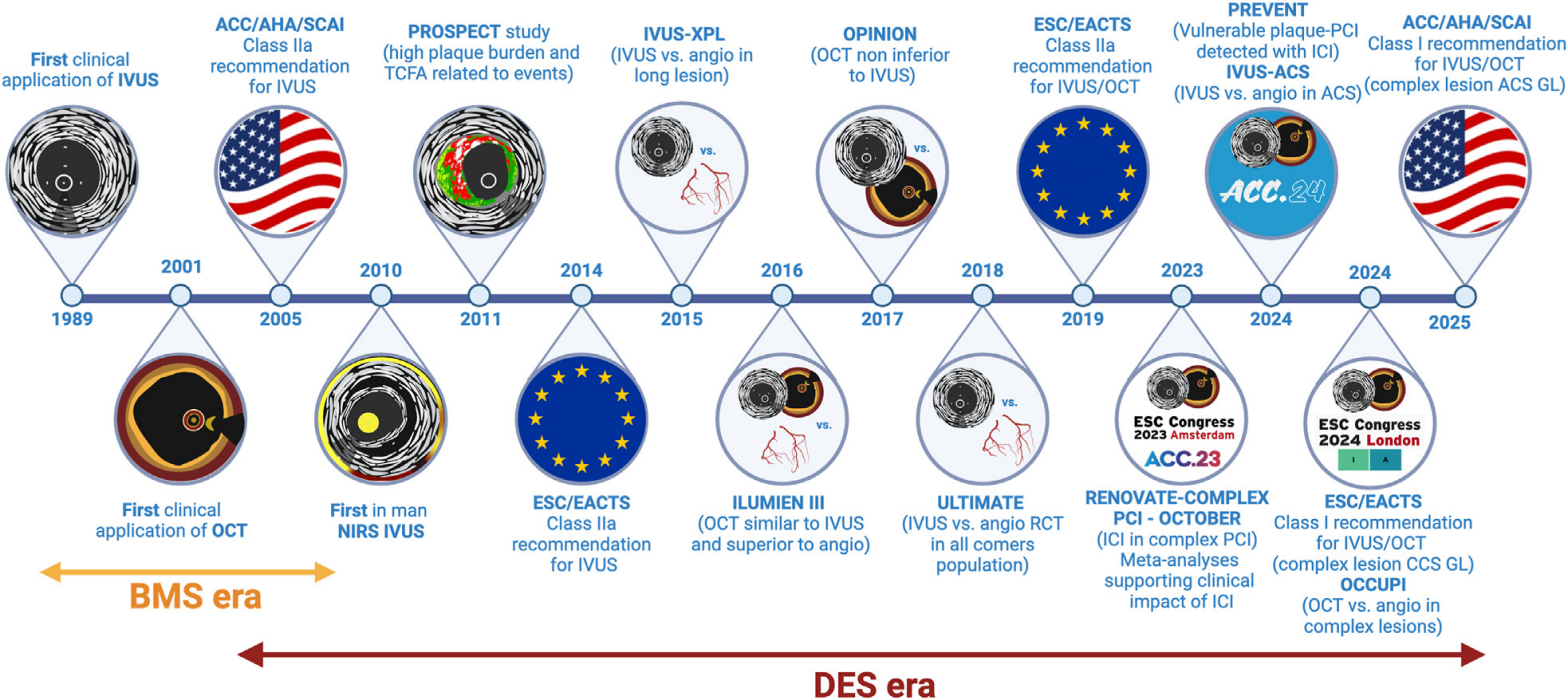
**Historical timeline of intracoronary imaging.**^1^^,^^2^^,^^4^^,^^9^, ^10^, ^11^^,^^18^^,^^21^^,^^23^^,^^25^^,^^33^, ^34^, ^35^, ^36^, ^37^, ^38^, ^39^, ^40^ ACC, American College of Cardiology; ACS, acute coronary syndrome; AHA, American Heart Association; BMS, bare-metal stent; CCS, chronic coronary syndrome; DES, drug eluting stent; EACTS, European Association for Cardio-Thoracic Surgery; ESC, European Society of Cardiology; GL, guidelines; ICI, intracoronary imaging; IVUS, intravascular ultrasound; OCT, optical coherence tomography; NIRS, near-infrared spectroscopy; PCI, percutaneous coronary interventions; SCAI, Society for Cardiovascular Angiography and Interventions.

Over the last 3 decades the use and applications of ICI have changed according to the evolution of interventional cardiology (Figure 1).^1^^,^^2^^,^^4^^,^^9^, ^10^, ^11^^,^^18^^,^^21^^,^^23^^,^^25^^,^^33^, ^34^, ^35^, ^36^, ^37^, ^38^, ^39^, ^40^ The first wave of studies with IVUS focused on in vivo assessment of coronary atheroma, providing valuable observations on phenomena like vessel remodeling during atherogenesis, along with differences in plaque structure in chronic and acute coronary syndromes.^41^, ^42^, ^43^, ^44^ With the arrival of bare metal stents (BMS) in the 1990s, IVUS demonstrated the importance of achieving adequate stent expansion and apposition to prevent stent thrombosis.^45^ The arrival of DES led to the hope that stent restenosis had been eventually resolved, casting doubts on the relevance of using IVUS for this purpose. However, growing awareness that stent failure, in particular stent thrombosis, had not disappeared with the arrival of DES led to a renewed interest in using ICI to guide PCI,^46^^,^^47^ with multiple RCTs, registries, and meta-analyses supporting the clinical value of ICI in multiple scenarios.^9^^,^^11^^,^^18^, ^19^, ^20^^,^^33^, ^34^, ^35^^,^^48^^,^^49^ Recently published American and European guidelines recommend ICI for performing PCI on anatomically complex lesions (including the left main stem) with a class I, level of evidence A.^21^^,^^25^ The American College of Cardiology (ACC) Interventional Council recently proposed the use of ICI as an essential adjunct to angiography for specific (generally complex) lesion subsets or any scenario where angiography may inadequately elucidate anatomy.^31^

## Uptake of ICI in the real world

**Figure 2:**
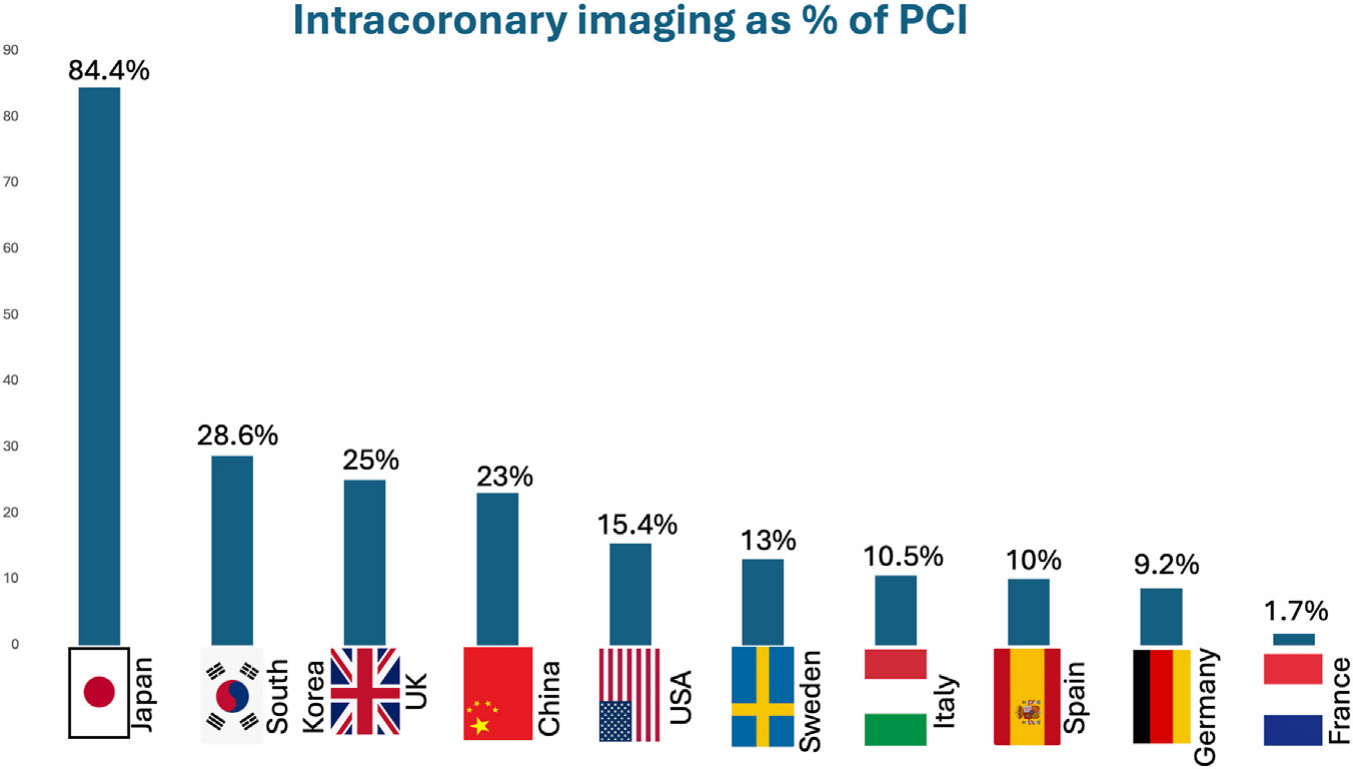
**Intracoronary imaging as percentage of percutaneous coronary intervention.**^50^, ^51^, ^52^, ^53^, ^54^, ^55^, ^56^, ^57^, ^58^, ^59^ PCI, percutaneous coronary intervention.

Despite all above-discussed evidence and recommendations, the global utilization rate remains low^50^, ^51^, ^52^, ^53^, ^54^, ^55^, ^56^, ^57^, ^58^, ^59^ (Figure 2). An international survey on ICI for interventional practice^60^ reported overall consistent results, with a very high penetration of ICI in Japan, while other regions had a more selective use of imaging. Use of ICI varies enormously between not only countries but also hospitals and operators within the same institution. Data regarding the uptake of ICI mostly stems from national or state registries and industry databases. Comprehensive data from most countries and tracking of temporal trends of the utilization of ICI in different countries are generally wanting. Detailed information of uptake of ICI in the real world is available in the Supplementary Material.

## Reasons behind successful ICI uptake

Japan’s early and long-standing uptake of ICI has been attributed to 2 key factors: (1) an early interest in IVUS technology, which was introduced in Japanese hospitals for clinical research as early as 1993 and soon enabled the application in clinical practice of validated quantitative, outcome-based measurements such as minimum stent area to predict future revascularization; and (2) a reimbursement accreditation framework that facilitates the approval of new medical devices based on a noninferiority criterion rather than requiring superiority in all aspects, leading to reimbursement of IVUS in Japan in 1994. This approach contrasts from that of many other countries, where reimbursement policies often require extensive cost-effectiveness and clinical utility data before approval, creating additional barriers to adoption.^61^

A recent study from Veterans Affairs hospitals in the United States reported an increase in ICI utilization, reaching 43.1% in 2022 with a notable inflection point in 2018 probably related to the results of clinical trial data and endorsement of scientific societies.^62^ However, the variability in ICI use was mainly driven by hospital and physician rather than PCI complexity or patient-level characteristics, highlighting the separate role of lack of evidence and physician-level factors in ICI uptake.

## Analysis of factors influencing uptake of ICI

In the following sections, we provide an overview of the factors that may contribute to the low uptake and high heterogeneity in utilization of ICI in most countries highlighted by the above-discussed registries.

### Factors affecting acceptability of ICI

**Figure 3:**
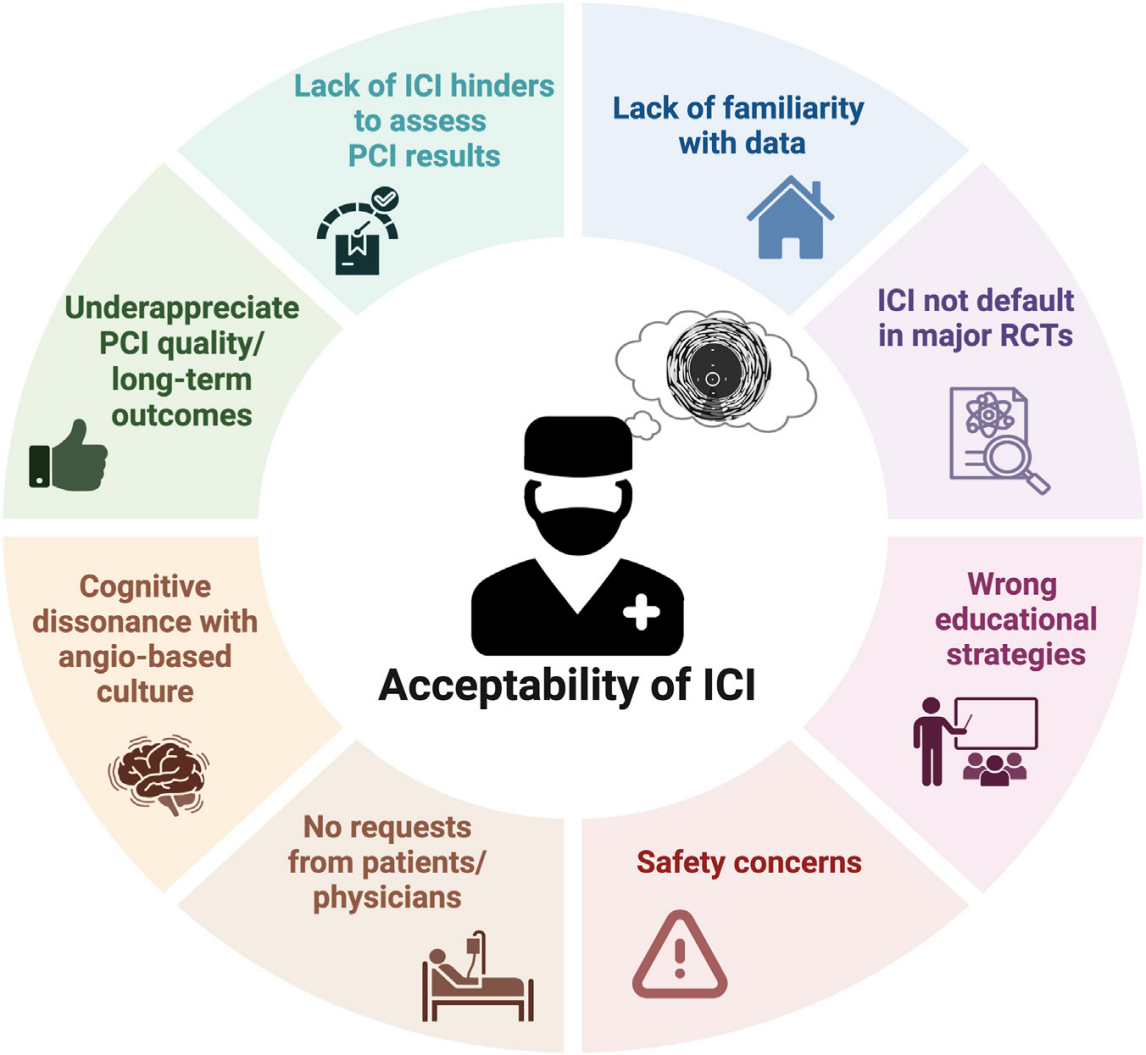
**Identified key factors influencing acceptability of intracoronary imaging.** ICI, intracoronary imaging; PCI, percutaneous coronary intervention; RCT, randomized controlled trial.

The key question in analyzing acceptability of ICI is why so many interventionalists are inadequately motivated to use it. At a difference with emerging technologies, IVUS and OCT have been available for decades and no longer can be envisaged as emerging or new technologies. Therefore, acceptability of ICI by operators is likely to be influenced by their exposure to educational events, scientific articles, and dedicated documents on the topic, as well to interactions with colleagues with a personal experience in using ICI. Further, we discuss potential aspects affecting such behavioral intention (Figure 3).

#### Underappreciation of the link between suboptimal PCI and long-term patient outcomes

A driving force to improve PCI quality is awareness of the consequences of a suboptimal intervention. Because clinical events related to suboptimal PCI typically manifest in the mid-term and long-term, interventional cardiologists rarely witness (or are notified) of long-term cardiac events occurring in intervened patients. Operators may be reinforced in believing that their PCI procedures are always optimal despite not using ICI when they do not witness long-term PCI-related events. Eg, before an episode of early stent thrombosis, the first question raised may be related to adherence to dual antiplatelet therapy rather than whether optimal stent implantation had been performed at the index PCI. The temporal dissociation between the immediate PCI result and the subsequent outcomes may further decrease the perceived usefulness of ICI.

#### Lacking ICI availability hinders the possibility of benchmarking and assessing PCI results

Closely related to the previous topic, not having the opportunity to access to ICI impedes the operators to benchmark their PCI results through the lens of ICI—which otherwise might generate awareness of suboptimal PCI results despite what may appear to be a sound angiographic result.

#### Lack of familiarity with data supporting the value of ICI

The way supportive evidence for ICI is perceived, evaluated, and assimilated is influenced by factors such as personal experience, training, and biases.^63^ Since ICI has been available for decades, gauging its value can be a challenge for many physicians, as some studies are less relevant (eg, those from the BMS era), while other focus on specific PCI niches. As a result, many interventional cardiologists may be unfamiliar or have an incomplete understanding of more recent evidence supporting the use of ICI in specific anatomical or clinical subsets.

#### ICI not being consistently part of a default PCI strategy in major clinical trials

Use of ICI has not been mandatory in most contemporary PCI trials. This may generate the false impression that acute or long-term PCI-related events are extremely rare when using contemporary device technologies and that, therefore, patient outcomes will not be further improved by using ICI. Rapid changes in the PCI landscape may also explain why ICI can be perceived as no longer needed (eg, over the transition from BMS to DES).

#### Misleading educational strategies deterring interventionalists from ICI use

By assigning bold opposing positions in controversy/debate sessions, anti-imaging statements can be viewed as indicating a lack of consensus on its clinical value. Educational models portraying ICI as something that requires the discourse of an expert analyst (quite frequent in live case transmissions) may intimidate the average interventionalist and generate an unfavorable perception of ICI’s ease of use.

#### Safety concerns of ICI

Operators may mistakenly perceive that incorporating ICI into procedures escalates procedural risks. In a contemporary international survey, 9.5% of respondents identified risk for procedural complications as the main factor limiting clinical use of ICI in clinical practice.^60^ This misconception could stem from concerns about prolonging procedural time or encountering technical challenges associated with imaging modalities.

#### No request for ICI use from patients and referring physicians

Acceptability of a PCI tool by operators is influenced by the perception that referring physicians and patients have on its clinical relevance or of being a marker of procedural quality. While dedicated surveys on this topic are lacking, it is quite likely that most referring physicians and patients are not aware or have not been informed on the impact of ICI guidance on PCI outcomes, including lower mortality.

#### Cognitive dissonance in an angiography-based culture

Psychological research has shown that opinions and attitudes tend to exist in clusters that are internally consistent. When consistency is challenged (dissonance), an individual will try to reduce it and avoid situations and information that would likely increase such dissonance.^64^ Adding new information from ICI, on top of what we have generationally grown accustomed to from angiography, may create dissonance. The challenge of reconciling existing practices with new conflicting information may trigger 3 psychological responses linked to cognitive dissonance: avoidance, delegitimization, and limiting its impact.

Interventional cardiologists trained only on angiographic PCI guidance may experience cognitive dissonance when prompted to uptake ICI, as it somehow challenges or questions their way of proceeding for years. Frustration arising from unsuccessful first ICI experience may also polarize interventionalists against further ICI use. Far from dissipating cognitive dissonance, unidimensional educational models on ICI, which are leveraged on supportive evidence and clinical practice guideline recommendations may instigate even further this psychological response.

### Factors affecting acceptance of ICI by interventional cardiologists

**Figure 4:**
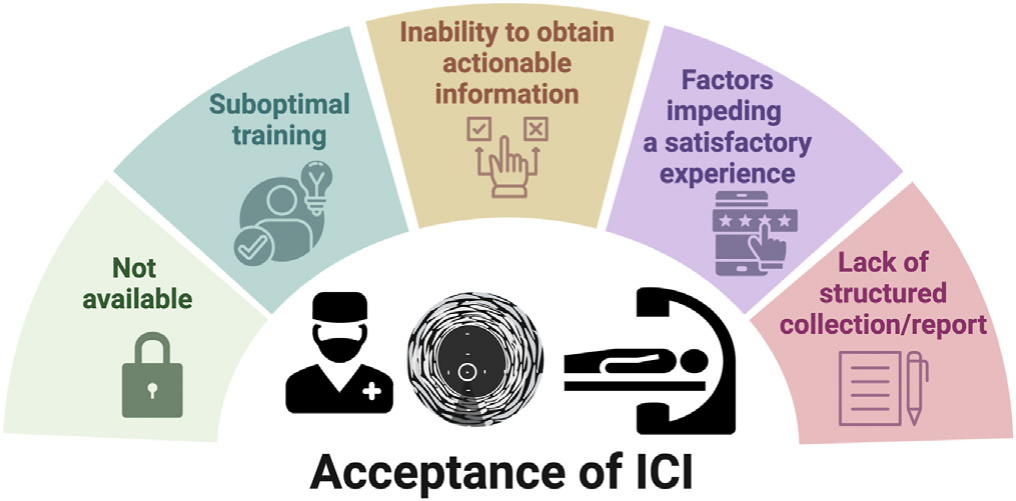
**Identified key factors influencing acceptance of ICI.** ICI, intracoronary imaging.

Successful incorporation of ICI is modulated by the personal experience of the operator (Figure 4). The key question in considering acceptance is what has contributed to a positive or negative operator experience in using ICI. Identified key factors influencing acceptance are described further.

#### Nonavailability of ICI

A fundamental cause of low acceptance of a technology is lacking personal experience with it. Only by getting firsthand experience can operators see the benefits of ICI and build confidence in its value. Having direct experience also implies that operators can receive customized onsite training and, which can significantly contribute to acceptance, which facilitates further steps toward adoption of the technology.

#### Suboptimal training in image interpretation

A 2020 survey revealed that a substantial proportion of interventional cardiology fellows-in-training initially reported themselves as experts or with sufficient experience in intracoronary physiology, IVUS, and OCT. However, among those who report such training, in fact, very few declared to be independent in all components of performance and interpretation required for clinical practice.^65^ In another survey, almost 60% of respondents wanted to increase appropriate ICI use and engage in online training.^66^ Lack of training and procedural lengthening associated to ICI were more common concerns for operators with fewer years of experience (<5 years), which likely accounts, in part, for low use of ICI in those with lower experience compared with that in more experienced operators.^60^

#### Inability to obtain actionable information from ICI

For many interventionalists, it may be unclear when and for whom ICI is indicated. The sheer volume of information encoded in intracoronary images can be overwhelming for operators embarking on a PCI during daily clinical practice. Without proper guidance or training, physicians may struggle to discern what matters most and how to extract and interpret relevant information. This challenge is also compounded by the dynamic nature of catheterization laboratory procedures, which require quick online and real-time interpretations.

### Factors impeding building a satisfactory experience in ICI

Several factors may impede building a satisfactory experience with ICI in the catheterization laboratory:

1. Heavy case load: Incorporating ICI into the workflow in catheterization laboratories with packed case lists can be challenging, as the learning curve is associated with more time required for preparing and performing ICI studies.
2. Dependence of external support: In some countries, ICI procedures are often performed only when a company representative is physically present to operate the machine assisting with image interpretation. This reliance on external support may hinder operators from independently gaining hands-on experience, limiting their confidence and familiarity with the technology.
3. Lack of engagement of NAPs: Although NAPs are not the prescribers and decision-makers in the use of ICI solutions, their active engagement plays a very important role in building a satisfactory experience and a favorable perception of ease of use of ICI technologies. This will be discussed in detail later when analyzing factors influencing adoption of ICI.
4. Technical failures or unsuccessful integration of ICI system in the catheterization laboratory: seamless integration of ICI can be hampered by technical issues, which, if not overcome promptly, may trigger resistance to further ICI use.

#### Lack of structured collection and reporting of ICI data

From a broad perspective, documenting in medical reports relevant data that underpin critical decisions contributes to robust reporting of an intervention. Yet, this is rarely the case in PCI even when ICI is used, probably because operators often focus on adhering to operating room schedules and ensure timely procedures. Deprioritizing reporting of ICI data may contribute to lower acceptance of this technology.

## Adoption of ICI

**Figure 5:**
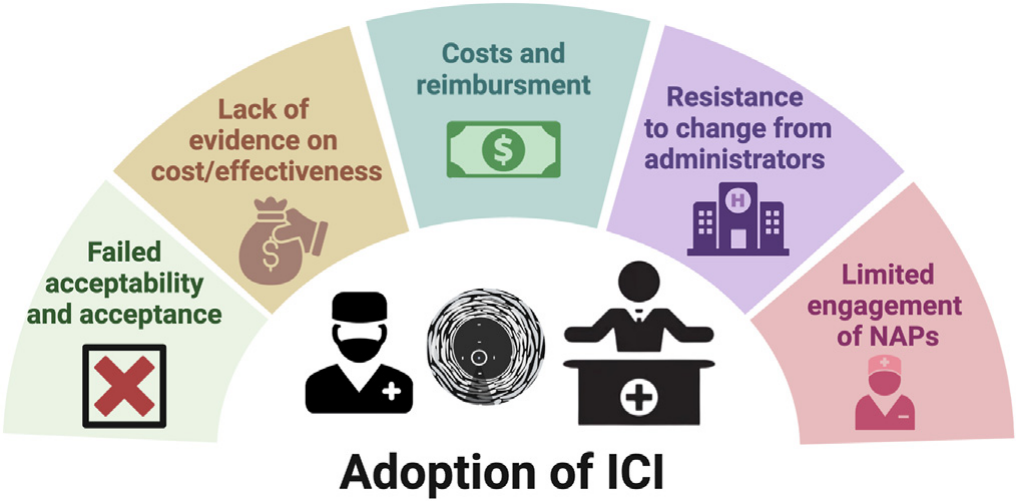
**Identified key factors influencing adoption of ICI.** ICI, intracoronary imaging; NAP, nurse and allied professional.

The ICI adoption process consists of several stages that begin with the acceptance of the technology by PCI operators and extends to other personnel involved in patient interventional management, culminating with the commitment of hospital administrators and health care systems (Figure 5). Some of the factors that may influence adoption are discussed further.

### Failed acceptability and acceptance

In the absence of a perceived usefulness and ease of use by the interventional team, it is unlikely that ICI will be adopted in a medical institution. Failed acceptability and acceptance of ICI may explain the high heterogeneity in ICI uptake noted between operators working in a single institution, which is most likely to occur in health care environments with autonomous interventionalists not constrained by departmental or service norms, leading to less uniform delivery of medical care.

### Limited evidence on the long-term cost-effectiveness of ICI

Assessing the cost-effectiveness of diagnostic methods like ICI is inherently more complex than evaluating treatments due to the nuanced nature of diagnostic benefits. In addition, cost-effectiveness data are not always well-understood by physicians and health care administrators. Detailed information about studies supporting cost-effectiveness of ICI can be found in Supplemental Material. This information gap is crucial, as robust data on long-term cost-effectiveness could mitigate the reluctance of health care providers.

### Costs and reimbursement issues

Device cost and lack of reimbursement are frequently reported barriers to utilization of ICI. Physician surveys have consistently demonstrated cost as a deterrent to ICI uptake. In the EAPCI-CVIT ICI survey, it was the primary barrier to use (66% of respondents), and in the recent UK ICI survey, just under 50% and around one-third of physicians reported upfront cost and a lack of demonstrated cost-effectiveness data, respectively, as core barriers to use.^60^^,^^66^

Reimbursement can be defined as the process by which clinicians or the hospital system are compensated for any costs associated with the device, including the device itself, the procedure, and any associated care settings. There are, however, many differences in the features of this process between countries and between health care models (eg, public or private). Due to this, establishing when reimbursement is a critical factor in ICI uptake can be challenging. Examples on the differences between reimbursement systems, and its impact on ICI uptake, are provided in the Supplementary Material.

### Resistance to change from hospital administrators or health care systems

Although hospital administrators and health care systems are not directly involved in the clinical application of ICI, their support and approval are essential for its widespread adoption. Resistance from these key decision-makers can significantly hinder the integration of ICI into routine clinical practice. Several reasons contribute to this resistance:

1. Financial constraints and budget priorities: Administrators may be reluctant to invest in ICI technologies due to the costs associated with equipment, training, and maintenance, particularly if the perceived return on investment is not immediately clear.
2. Unfamiliarity with clinical benefits: A lack of understanding of the clinical advantages (ie, reduced rehospitalization), and potential long-term cost savings associated with ICI utilization can lead to hesitancy in supporting its adoption.
3. Resistance to change: established protocols and workflows in hospitals can create a preference for maintaining the status quo, making it challenging to introduce new technologies like ICI.

### Resistance to change from catheterization laboratory nurses and technicians

Although NAPs are not the prescribers and decision-makers in the use of ICI solutions, their engagement in these strategies is crucial to the implementation and development of the technique. Although literature is relatively limited, we can identify several reasons for this problem.

One key issue is the lesser degree of involvement that NAPs often have in the procedure, particularly in tasks such as image acquisition and data interpretation. This limited participation can diminish their motivation for ICI utilization.

In low-volume centers, where opportunities to use ICI are infrequent, a lack of familiarity with device preparation and handling can develop. This creates a vicious circle, where reduced usage leads to decreased confidence and comfort with the technique (the less you do, the less comfortable you are). In particular, it can impact the confidence in interpreting imaging data and understanding the implications of ICI findings for procedural outcomes. The latter is often related to local catheterization laboratory policy and inadequate education on the latest developments in the field, including recent clinical trial results.

Additionally, organizational factors and issues for easy access to the devices within the catheterization laboratory can pose significant challenges. This point could appear as somehow trivial but represents a real bottleneck in the ICI-assisted PCI workflow. This is a special concern in institutions where the ICI devices are mobile stations that are not stored in the main operating room and are not easily accessible.

## Suggested actions to improve uptake of ICI

By considering all above-discussed reasons impeding broader utilization of ICI, the following actions are proposed to overcome identified obstacles and support wider adoption of ICI, as summarized in Table 1.

**Table 1:** Summary of proposed actions to overcome the identified obstacles and support wider adoption of ICI.

AI, artificial intelligence; ICI, intracoronary imaging; NAP, nurse and allied professional; PCI, percutaneous coronary intervention.

### Improving education and familiarity with evidence supporting ICI among interventional cardiologists

Education can be improved by incorporating insights garnered from surveys and educational frameworks that apply the Socratic method,^67^ which encourages thinking critically and generating ideas instead of relying on lectures and provision of direct information. A proposed example is available in the Supplementary Material.

Existing and evolving technologies, such as coregistration techniques^68^ and artificial intelligence (AI)-powered automated interpretation tools, may be of support for ICI interpretation by reducing the cognitive load on operators while enhancing efficiency and effectiveness.^69^ However, robust data supporting these educational approaches are still lacking. For example, machine learning automatic segmentation of IVUS images has shown a very good correlation with expert IVUS analysts, accurately selecting an appropriate balloon size in >90% of IVUS images to guide PCI.^70^ Furthermore, AI frameworks for automatic plaque characterization in OCT have also provided an excellent diagnostic accuracy reducing subjectivity in image interpretation and facilitating OCT quantification of plaque composition, with promising clinical applications.^71^

As these technologies continue to evolve, it is imperative to adequately prepare interventionalists, especially those less familiar with technological advancements, for their immediate clinical implementation through educational programs.

### Choice of educational models

The type of educational product and model may significantly influence the uptake of ICI. Many interventional cardiologists may be reluctant to use any ICI modality whose images they do not fully understand, often leading to reliance on angiography guidance. Education should not only promote ICI’s clinical benefits but also focus on improving image interpretation skills through structured learning strategies.

For instance, hands-on or case-based learning models may facilitate uptake of ICI more effectively than traditional evidence reviews or technical lectures. On the contrary, educational models portraying ICI as something that requires consultation with an expert analyst (quite frequently during live case transmissions) may intimidate the interventionalist interfering with ICI utilization.

Regular assessment of acquired skills, combined with workshops and online resources, can reinforce learning and track progress. The educational needs may be different for operators who use ICI only for a handful of cases as opposed to those who use it more regularly. Different educational modules might be developed for different PCI types or patient subsets. Therefore, developing an ICI compendium focused on various clinical scenarios, such as acute coronary syndrome, bifurcation lesions, calcific lesions, and chronic total occlusions, may provide a structured resource for learners to enhance their understanding and application of ICI.

As suggested by the VARK model, humans have different learning preferences, like visual (V), auditory (A), read/write (R), and kinesthetic (K), for acquiring and effectively processing information.^72^ Therefore, each trainee may have a different learning modality underscoring the need for dedicated and personalized curricula. It is foreseeable that education on ICI interpretation based on AI platforms will also take place in a near future.

The ACC Interventional Council recently proposed an approach for the clinical use of ICI, which involves programmatic recommendations at operator, institutional, and national levels. The authors highlight the role of professional societies in developing and offering lifelong training opportunities and competency tools. It also underscores the importance of national recommendations regarding training programs, which should expose trainees to an adequate volume of ICI in order to achieve an adequate competency.^31^ Examples of potential educational models are available in the Supplementary Material.

### Dissipating concerns on the safety of intracoronary instrumentation with ICI

Highlighting the safety of ICI imaging in future studies using these technologies may add to the already existent evidence on its safety. Specific safety protocols to mitigate the risk of complications associated with ICI in specific clinical scenarios may also be useful. Examples of the latter are avoidance of forceful crossing of a freshly implanted stent or interrogation of spontaneous coronary dissection if a diagnosis has been reached on angiographic grounds.

### Addressing reimbursement issues

Future RCTs in ICI should include in-parallel prospective health economic analysis, as undertaken in the RENOVATE–COMPLEX PCI RCT^73^ and currently being undertaken in the IMPROVE Trial.^74^ This approach, alongside active dissemination of existing health economic data to physicians, administrators, and health technology assessment boards, may help to facilitate improved understanding of the nonclinical longer-term benefits of ICI and dispel assumptions of increased cost as a barrier to utilization, particularly in complex patient and lesion populations. It will also provide the evidence required to facilitate health technology assessment approval and reimbursement across jurisdictions and different health care systems.

Payers may use clinical guidelines in deciding if an intervention is needed and how should be performed, taking this into consideration for reimbursement decisions. In this direction, endorsement of use ICI by clinical practice guidelines may influence payers in linking PCI procedures to the use of ICI guidance.

### Structured collection and reporting of ICI data in PCI reports

Incorporating detailed ICI data into the PCI report offers a comprehensive description of the procedure. Additionally, transparent documentation of ICI findings fosters accountability and trust among patients and colleagues, enhancing both the actual and perceived quality of the procedure performed.

However, a dissonance exists between various imaging end points among different clinical trials, and operators may feel uncertain about what data to report. An agreement regarding imaging end points that should be reported from the EAPCI, as recently performed by the CVIT, is needed.^28^

To facilitate an efficient report of these extensive data, an automated transfer of information from imaging workstations to PCI reports is suggested, akin to the process used for transthoracic echocardiogram reports. This approach can streamline the documentation process, ensuring consistency in reporting standards and potentially serving as a quality measure for the procedure performed. Moreover, the automated system simplifies the compilation of standardized reports, facilitating effective communication of imaging findings also to colleagues reviewing PCI reports.

Storage of ICI data are still a time-demanding and not fully automated process, which may hamper ICI uptake. Enhanced solutions such as the image-archiving and communications systems (PACS) are imperative to streamline these processes and improve efficiency in ICI utilization and reporting.^31^ Efforts from manufacturers should be directed toward developing these automated reporting systems also addressing current challenge regarding storage process. In Supplemental Material, we provide a suggested framework for structuring and reporting ICI data in PCI.

### Fostering engagement of catheterization laboratory nurses and technicians

A number of strategies to improve engagement of NAPs in the catheterization laboratory about using ICI techniques can be proposed. The interventional community should develop comprehensive education and dedicated training sessions for the NAPs on the importance and benefits of ICI. This process could be achieved through scientific meetings, workshops, or proctored sessions, which help them understand how ICI can improve patient outcomes and enhance the quality of care. Providing hands-on practice with ICI devices would allow NAPs to build confidence and familiarity with the equipment, making them more likely to embrace the techniques in their practice.

Open communication and dialog should also be encouraged, ensuring NAPs have the opportunity to express their concerns and address any misconceptions about these techniques. Creating a supportive environment fosters their engagement and comfort. Integrating ICI devices more seamlessly within the catheterization laboratory may decrease unneeded work by NAPs and increase time efficiency. Finally, identifying catheterization laboratory imaging champions within the NAPs staff, as recommended by the Society for Cardiovascular Angiography & Interventions (SCAI),^75^ can facilitate peer-to-peer support and guidance for the less experienced colleagues, favor intrateam education, and ultimately increase ICI uptake.

### Reducing cognitive dissonance

To reduce cognitive dissonance, educational efforts may need to focus on specific and actionable information obtained from ICI but missed by angiography. An example of this is the identification with ICI of procedural complications like unintended stent deformation.^76^ By institutionalizing the claim that additional information from ICI is often needed to overcome limits of angiography, cognitive dissonance may be reduced. On the contrary, by educating on well-defined, actionable information from ICI, we reduce cognitive dissonance and increase acceptance by enhancing its practicability and ease of use. Tackling cognitive dissonance thus stands at the intersection of acceptability and acceptance.

### Patient and referring cardiologist empowerment strategies

Social media or traditional media can be used to effectively communicate the clinical benefits of ICI in a clear and straightforward manner to patients, referring cardiologists, and the overall population. Sharing relevant ICI data or images with the PCI report may increase the perceived quality of the intervention by referring cardiologists and patients. It may also serve as a legal safeguard in the event of unwarranted complaints by providing documentation of the quality care delivered to address and treat the patient’s condition.

## Conclusions

There are a multiplicity of factors influencing ICI uptake. Available evidence and clinical practice guidelines recommendations supporting the use of ICI constitute an urgent call to change the current landscape of interventional cardiology practice, which is characterized by poor acceptance, acceptability, and adoption of ICI. While additional factors influencing ICI uptake are likely to exist, these problems are not insurmountable, and specific strategies need to be implemented in order to expand the practice of ICI, a data-driven PCI quality indicator.
