# Supplementary material for: Factors Contributing to Low Utilization of Intracoronary Imaging in Clinical Practice: A White Paper
Source: J Soc Cardiovasc Angiogr Interv. 2025 May 20;4(7):103607. doi: 10.1016/j.jscai.2025.103607 (PMC12418432; doi:10.1016/j.jscai.2025.103607)
Supplement: Supplementary Material [file mmc1.pdf]

SUPPLEMENTARY MATERIAL

Supplementary Table 1. Commercially available intracoronary imaging systems in 2025.

Intracoronary imaging modality	Source of image	Commercially available system
IVUS	Ultrasound	• AVVIGO+ Multi-Modality system - OptiCross catheter family (Boston Scientific, United States),<br>• IntraSight/SyncVision systems – Eagle Eye Platinum and Refinity catheters [Philips (formerly Volcano), United States and Netherlands),<br>• ACIST HDi system – Kodama catheter (ACIST, United States),<br>• Visicube system – AltaView/Navifocus catheter (Terumo, Japan),<br>• VivoHeart system - True Vision catheter (Insight Lifetech, China).
OCT/OFDI	Infrared light	• OPTIS NEXT system (Ultreon software)/ OPTIS system – Dragonfly Opstar/Optis catheter (Abbott Vascular, United States),<br>• Lunawave system - FastView catheter (Terumo, Japan),<br>• Gentuity system – Vis-Rx catheter (Nipro, Japan),<br>• Aurios OCT System – Benetis catheter (Dotter, South Korea).
Hybrid	Ultrasound / infrared light / spectroscopy	• OCT/IVUS: Novasight Hybrid system and catheter (Conavi Medical, Canada),<br>• OFDI/IVUS: Dual Sensor (Terumo, Japan),<br>• NIRS/IVUS: Makoto system – Clarispro/Dualpro catheter [Nipro, Japan (formerly Infraredx, United States)],<br>• OCT/NIRS: Hypervue system – Starlight catheter (Spectrawave, United States),<br>• IVUS/OCT: Panovision hybrid system (Panovision Co., Ltd, China).

*Note: This table presents an overview of commercially available intracoronary imaging systems as of February 2025. Due to the evolving nature of medical device, this list may not be exhaustive.

Uptake of ICI in the real world

In France the reported overall uptake of ICI was 1.7% of all PCI (1), with variability between different cath labs ranging from 0 – 9.5%. In Sweden, the overall mean utilization of ICI reported in 2023 was 13% (2), with ICI utilization ranging from 3% to 26%. However, ICI use in Sweden seemed to increase over time, since ICI use was 5% in 2013. Also, in Sweden ICI has been implemented as a quality indicator in the nationwide SWEDEHEART registry. The United Kingdom (UK) has the highest reported utilization rate of ICI in Europe. The British Cardiovascular Intervention Society (BCIS) analysis of over 85,000 PCI cases performed between 2022 and 2023 in England and Wales, demonstrated an overall utilization rate of ICI in 25% of cases, with 26% utility in complex PCI cases and 74% use in unprotected left main (3). Industry databases based on sales statistics although not publicly available suggest an overall uptake of ICI in Europe of about 10-15% with significant variation between different countries.

The situation in the United States (US) shows similar trends than in Europe. Data from the Blue Cross Blue Shield of Michigan Cardiovascular Consortium (BMC2) registry showed a mean use of ICI in 16.6% of PCI procedures between 2019 and 2021, increasing from 7% to 23% over the study period. Although this represents a rise compared to the previously reported 5%-6% ICI utilization in US PCIs between 2004 and 2017, overall uptake remains relatively low. Similarly, there was a very high degree of variability in the use of ICI between different hospitals, ranging from <1% at several hospitals to nearly 75% at others (4).

Japan has a very high degree of ICI use in PCI. A report from a multicenter PCI registry of consecutive patients treated with PCI between 2008 and 2014 demonstrated 84.8% use of ICI (5). In Korea, the utilization rate of ICI was reported to be 28.6% in a 2014 cohort study, with higher usage at high-volume centers (6). In China, the mean rate of ICI use is approximately 23%, varying from 13% to 45% depending on geography. Notably, 90% of ICI use in China involves IVUS guidance (7).

Studies on cost-effectiveness of intra-coronary imaging

As mentioned in the main text of the document, device cost and lack of reimbursement is a frequently reported barrier to utilization of intracoronary imaging (ICI) (8,9). In contrast to the crude expense of the ICI catheter, cost utility

and effectiveness are challenging to accurately capture, sparsely available and poorly understood by physicians and technology appraisers.

Early studies of cost-effectiveness originated from Denmark and Germany in the bare-metal stent (BMS) era, where Gaster and colleagues analyzed in 108 all-comers randomized to intravascular ultrasound (IVUS) or angiography – guided percutaneous coronary intervention (PCI) with satisfactory PCI defined as per MUSIC criteria. IVUS was cost-saving at 6 – months driven by a reduction in repeat revascularizations for in-stent restenosis with benefit increasing to 2-year and maintained at 4-year follow-up (10,11). Mueller et al. observed similarly in 2003, with a significant reduction in 2-year major adverse cardiovascular events (MACE) [80 v 69% IVUS vs coronary angiography, p<0.04] primarily driven by target lesion revascularization (TLR). With an incremental cost-effectiveness ratio (ICER) of $1417 per MACE event-free survival, IVUS was cost-saving over 50% of the time (12).

These analyses were undertaken in randomized controlled trial's (RCT) utilizing BMS. With the advent of drug-eluting stent (DES), we saw a natural decline in restenosis and TLR, and a decade of dormancy followed regarding evaluation of cost effectiveness. More recently, driven by increasing evidence supporting reduction in MACE, with use of ICI in DES PCI, there has been a resurgence of interest in health economic assessment, centered around identifying optimal patient and lesion cohorts and breaking down barriers to ICI use.

In 2016 Alberti and colleagues modelled cost effectiveness assuming limited benefit of IVUS to only 12 -months. An ICER of 9624 euros, well below the Italian willingness to pay [WTP] threshold of 25,000 euros was observed. On lifetime modelling, IVUS was also found to be cost saving. Incremental cost benefit was seen in cohorts at high-risk of stent failure [acute coronary syndrome (ACS), renal failure and diabetes]. IVUS was also the dominant strategy in supplementary analyses on everolimus eluting DES consolidating device benefit for TLR reduction in 2nd generation DES – PCI (13). Similar was seen in a health economic analysis of IVUS – guided DES implantation in Australia. Modeling a lifetime horizon, an ICER of 17,539 Australian dollar (AUD), well below the WTP of 50,000 AUD was obtained. In exploratory sub-group analysis, cost – effectiveness was greatest among left – main stem and complex coronary

lesions, consistent with clinical outcomes of reduced MACE observed in these lesion cohorts in large RCTs (14).

In the United Kingdom (UK), utilizing a National Institute for Health and Care Excellence (NICE) recommended health economic modelling strategy, IVUS in an ACS population was 100% cost-effective at a UK WTP of £20,000. ICERs well below the health technology assessment (HTA) device approval threshold for cost-effectiveness, of £30,000, were observed in ST Elevation Myocardial Infarction (STEMI) [£3649] and non- ST Elevation Myocardial Infarction (NSTEMI) / unstable angina [£5706] cohorts with authors concluding cost to no longer be a credible barrier to utilization (15).

Finally, following from observational and population – modeled analyses, the RENOVATE – COMPLEX PCI RCT most recently reported its in-parallel health economic analysis findings of ICI use in complex coronary disease. ICI emerged the dominant strategy in 70% of iterations, generating cost neutrality at 5 – years and a cost-saving, negative ICER of $9063 on lifetime modeling with significant improvement in quality-of-life outcomes (16).

Differences in reimbursement systems

As discussed elsewhere in this document, reimbursement can be defined as the process by which the healthcare provider is compensated for any costs associated with the device, including the device itself, the procedure, and any associated care settings. Differences in the features of this process between countries and between healthcare models must be taken into account when assessing the impact of reimbursement on ICI uptake.

For example, in the United States, most hospitals are reimbursed through Diagnosis-Related Groups (DRGs), which provide a fixed payment for an episode of care rather than individual services. This system does not account for the higher resource utilization associated with ICI, creating a possible incentive for repeat procedures. As a result, hospitals typically bear the upfront incremental costs of ICIs, while third-party payers benefit from the downstream advantages of these treatments.

In the UK the Clinical Commissioning Groups and the NICE play a key decision makers in adopting a medical technology. Classification and reimbursement of healthcare services in the UK the NHS uses a system of Health Resource Groups

(HRGs), analogue to DRGs. Hospitals claim reimbursement from clinical commissioning groups (CCGs) based on HRG codes. Healthcare providers are paid for each patient seen or treated, taking into account the complexity of the patient's healthcare needs.

Alternatively, in Spain reimbursement of medical devices in Spain involves obtaining market authorization and approval from relevant health authorities. Main decision makers in the reimbursement process are HTA organizations. In Spain's 17 autonomous regions are responsible for delivering and financing healthcare, which includes determining their own budgets and reimbursement guidelines. Rather than a traditional reimbursement system, Spain operates under a funding framework where hospitals receive funding through a global budget. The allocation of these budgets is managed by the health authorities of the Autonomous Communities.

Role of reimbursement in driving ICI uptake

Reimbursement policies play a pivotal role in driving ICI uptake. A notable example comes from Poland, where an analysis of the national database from 2014 to 2021 showed a significant increase in ICI uptake, rising from 0.81% in 2014 to 4.83% in 2021—an almost sevenfold increase primarily attributed to changes in reimbursement policies (17). Additionally, the 2023 report from the Polish Cardiac Society indicated a further rise in ICI uptake compared to 2022 (18). However, despite this progress, overall utilization remains below 10%, highlighting that while reimbursement is a key facilitator, other barriers continue to limit widespread uptake of ICI, as discussed throughout this manuscript.

Examples to improve education and to guide the choice of adequate educational models

As mentioned in the main manuscript, education represents a key aspect to be addressed to overcome barriers to ICI uptake.

Here we report a survey example which may be helpful to address this pivotal aspect.

Initially, an evaluation of the level of competence in images interpretation should be conducted among interventional cardiologists or fellows-in-training. Subsequently, the framework would involve the application of ICI to real-world

clinical cases, utilizing angiographic dilemmas which illustrate the clinical utility of such modalities. Conducting a follow-up survey on the skills acquired following a dedicated educational curriculum in ICI interpretation would also be beneficial and provide valuable insights into the respondent's learning curve and self-evaluation aimed at improving the adoption of ICI.

Education in interventional cardiology involves a balance between volume and quality. Establishing a specific number of mandatory ICI procedures is an appropriate educative approach for developing uniform training standards across centers. However, volume is not the only issue to be addressed, as the quality of information gained from an ICI procedure is high.

A proposal for fellows-in-training on mandatory use of ICI in first PCI (e.g. 20 to 50) may be considered. In fact, initial exposure allows trainees to grasp the wealth of information obtained from such imaging modalities and, for instance, to appreciate the extent of atherosclerotic disease, which may not be fully evident through visual estimation with coronary angiography alone.

Another potential issue to be addressed is a lack of confidence if exposed to a non-protected environment without an expert/senior colleague available to help in interpreting intra-coronary images. To tackle this challenge, a possible educational model may involve achieving independence in a protected environment with expert assistance during meetings/workshops where trainees may feel more comfortable expressing their thoughts and concerns. Eventually, the ultimate goal is to attain the maximum level of independence in ICI, enabling interventional cardiologists to teach other colleagues/fellows how to perform and interpret it effectively. For instance, implementing training with real-time digital feedback on feature identification and measurements may be beneficial. Indeed, an augmented interactive OCT curriculum has demonstrated a significant impact on naïve readers compared to a standard approach with a self-directed didactic and consensus statement-based study (19).

Finally, since no data are currently available to support age-related disparities in ICI utilization, it is crucial to gather quantitative evidence. This would help emphasize the need for comprehensive training across all age groups, as ICI proficiency is not solely a matter concerning early-career interventional cardiologists.

Supplementary Figure 1. Suggested framework for structuring and reporting intracoronary imaging data.

**Institution**	**Hospital Name**<br>**Department of Cardiology Unit**<br>P.o.box<br>Tel: +

**Given Name:**		**Family Name:**	
**Date of Birth:**	**Age:**	**Sex:**	**Hospital ID:**
**Referring Physician:**		**Reason for ICI:**	

Intracoronary imaging (ICI) Report

**ICI Modalities**			
Type of ICI performed	IVUS ☐	OCT ☐	Hybrid ☐
ICI system used	*ICI pullback speed*		Manual ☐ Automatic ☐
**Vessel evaluated**			

Vessel	LM ☐	LAD ☐	LCx ☐	RCA ☐
Location	Proximal ☐	Mid ☐	Distal ☐	

**Pre-PCI Assessment**			
Morphology	Fibrous ☐	Calcified ☐	Lipid-rich ☐
Calcium Yes ☐ No ☐	Arc (°)	Thickness (mm)	Lenght (mm)
Vulnerability Yes ☐ No ☐	*Thrombus* Yes ☐ No ☐	*Rupture* Yes ☐ No ☐	*Erosion* Yes ☐ No ☐
Length (mm)			
Reference			

Lumen diameter and area	Proximal	(mm) (mm²)	Distal	(mm) (mm²)	
Vessel diameter and area	Proximal	(mm) (mm²)	Distal	(mm) (mm²)	

Minimum lumen diameter (MLD) (mm)		*Minimum lumen area (MLA)* (mm²)	
Mean lumen area (mm²)	*Percentage area stenosis (%AS)* %	*Mean lumen diameter* (mm)	*Percentage diameter stenosis (%DS) (%)*

Plaque burden (%)	*Lesion vessel area* (mm²)	*Remodeling*

**PCI guidance**				
Plaque preparation	Balloon ☐	Atherectomy ☐	IVL ☐	Laser ☐
Stent/balloon sizing	Diameter (mm)	Length (mm)		

**Post-PCI Assessment**		
Malapposition Yes ☐ No ☐	*Max malapposition length* (µm)	
Reference lumen area	*Proximal* (mm²)	*Distal* (mm²)
Mean reference lumen area (mm²)	*Minimum stent area (MSA)* (mm²)	*Stent expansion (%)*
Residual stenosis (>50%) Yes ☐ No ☐	*Edge dissections* Yes ☐ No ☐	
Optimization Yes ☐ No ☐	*Post-dilatation* Yes ☐ No ☐	*New stent* Yes ☐ No ☐

**Images (optional)**
Inclusion of representative images captured during the procedure, such as IVUS or OCT cross-sectional images, longitudinal views, and 3D reconstructions.<br>*Annotation of key findings on the images.*
**Final report:**

Examining physician: **Date:**

Abbreviations: ICI: intracoronary imaging, IVL: intravascular lithotripsy; IVUS: intravascular ultrasound; OCT: optical coherence tomography; MLA: minimun lumen area; MLD: minimum lumen diameter; MSA: minimum stent area; PCI: percutaneous coronary intervention; 3D: three-dimensional; %AS: percentage area stenosis; %DS: percentage diameter stenosis

Supplementary Figure 2. Cost-effectiveness of intracoronary imaging (10–16).

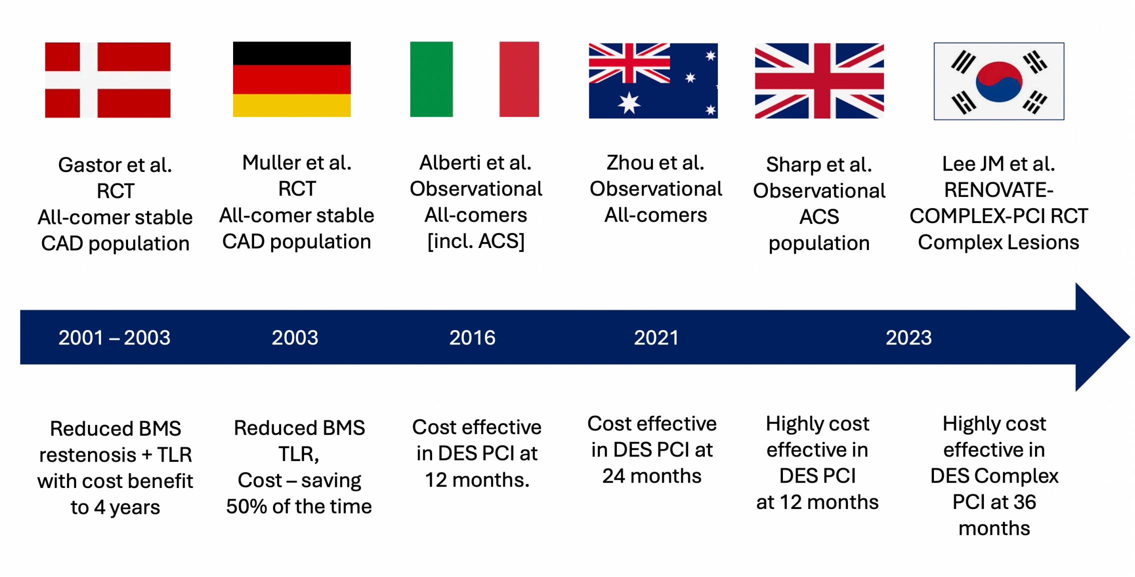

Abbreviations: ACS, acute coronary syndrome; BMS, bare metal stent; CAD, coronary artery disease; DES, drug eluting stent; ICI, intracoronary imaging; PCI, percutaneous coronary interventions; RCT, randomized clinical trial; TLR, target lesion revascularization

Supplementary references

1. Rangé G, Hakim R. [Penetration of endocoronary imaging in France (data from France PCI): The French exception]. Ann Cardiol Angeiol (Paris). 2022 Dec;71(6):350–5.

2. von Koch S von, Bergman S, Andell P, et al. Intracoronary Imaging of Proximal Coronary Artery Lesions – A Nationwide Lesion-Level Analysis From SCAAR. J Soc Cardiovasc Angiogr Interv. 2023;2(3).

3. https://www.nicor.org.uk/national-cardiac-audit-programme/angioplasty-audit-napci.

4. Madder RD, Seth M, Sukul D, et al. Rates of Intracoronary Imaging Optimization in Contemporary Percutaneous Coronary Intervention: A Report From the BMC2 Registry. Circ Cardiovasc Interv. 2022 Oct;15(10):e012182.

5. Kuno T, Numasawa Y, Sawano M, et al. Real-world use of intravascular ultrasound in Japan: a report from contemporary multicenter PCI registry. Heart Vessels. 2019 Nov;34(11):1728–39.

6. Jang JS, Han KR, Moon KW, et al. The Current Status of Percutaneous Coronary Intervention in Korea: Based on Year 2014 Cohort of Korean Percutaneous Coronary Intervention (K-PCI) Registry. Korean Circ J. 2017 May;47(3):328–40.

7. Yang L, Yaling H. Use of intravascular imaging guidance in percutaneous coronary intervention in China. Clin J Med Off. 2023;51:995–9.

8. The UK Intracoronary Imaging Survey – An Evaluation of Perceptions, Barriers, Education and Training – pending publication.

9. Koskinas KC, Nakamura M, Räber L, et al. Current use of intracoronary imaging in interventional practice - Results of a European Association of Percutaneous Cardiovascular Interventions (EAPCI) and Japanese Association of Cardiovascular Interventions and Therapeutics (CVIT) Clinical Practice Survey. EuroIntervention. 2018 Jul 20;14(4):e475–84.

10. Gaster AL, Slothuus U, Larsen J, Thayssen P, Haghfelt T. Cost-effectiveness analysis of intravascular ultrasound guided percutaneous coronary intervention versus conventional percutaneous coronary intervention. Scand Cardiovasc J SCJ. 2001 Mar;35(2):80–5.

11. Gaster AL, Slothuus Skjoldborg U, Larsen J, et al. Continued improvement of clinical outcome and cost effectiveness following intravascular ultrasound guided PCI: insights from a prospective, randomised study. Heart. 2003 Sep;89(9):1043–9.

12. Mueller C, Hodgson JMB, Schindler C, Perruchoud AP, Roskamm H, Buettner HJ. Cost-effectiveness of intracoronary ultrasound for percutaneous coronary interventions. Am J Cardiol. 2003 Jan 15;91(2):143–7.

13. Alberti A, Giudice P, Gelera A, et al. Understanding the economic impact of intravascular ultrasound (IVUS). Eur J Health Econ HEPAC Health Econ Prev Care. 2016 Mar;17(2):185–93.

14. Zhou J, Liew D, Duffy SJ, et al. Intravascular Ultrasound Versus Angiography-Guided Drug-Eluting Stent Implantation: A Health Economic Analysis. Circ Cardiovasc Qual Outcomes. 2021 May;14(5):e006789.

15. Sharp ASP, Kinnaird T, Curzen N, et al. Cost-effectiveness of intravascular ultrasound-guided percutaneous intervention in patients with acute coronary syndromes: A UK perspective. Eur Heart J Qual Care Clin Outcomes. 2023 Dec 18;qcad073.

16. Hong D, Lee J, Lee H, et al. Cost-Effectiveness of Intravascular Imaging-Guided Complex PCI: Prespecified Analysis of RENOVATE-COMPLEX-PCI Trial. Circ Cardiovasc Qual Outcomes. 2024 Mar;17(3):e010230.

17. Januszek R, Siudak Z, Malinowski KP, et al. Factors determining the frequency of optical coherence tomography and intravascular ultrasound use in patients treated with percutaneous coronary interventions in recent years: Analysis based on a large national registry. Kardiol Pol. 2023;81(10):969–77.

18. Siudak Z, Grygier M, Tomaniak M, et al. Interventional cardiology in Poland in 2023. Annual summary report of the Association of Cardiovascular Interventions of the Polish Cardiac Society (AISN PTK) and Jagiellonian

University Medical College. Postepy W Kardiologii Interwencyjnej Adv Interv Cardiol. 2024 Dec;20(4):379–81.

19. Kassis N, Weber JR, Adams W, et al. Immersive educational curriculum on intracoronary optical coherence tomography image analysis among naïve readers. BMC Med Educ. 2022 Oct 12;22(1):719.
